# The Detection of Activities Occurring Inside Quick Service Restaurants That Influence Air Quality

**DOI:** 10.3390/s22114056

**Published:** 2022-05-27

**Authors:** Andrzej Szczurek, Andi Azizah, Monika Maciejewska

**Affiliations:** Faculty of Environmental Engineering, Wroclaw University of Science and Technology, Wybrzeże Wyspiańskiego 27, 50-370 Wroclaw, Poland; andrzej.szczurek@pwr.edu.pl (A.S.); 257751@student.pwr.edu.pl (A.A.)

**Keywords:** indoor air quality, sensing, pattern recognition

## Abstract

Our attention was focused on the identification of activities affecting air quality, which occur in quick-service restaurants (QSR). The work was based on a measurement study of selected kebab stores in the Polish city of Wrocław. It demonstrated that activities taking place in kebabs altered air quality. The associated changes in air parameters such as temperature, relative humidity, CO_2_ concentration, and the content of volatile organic compounds could be detected by utilizing a simple, multi-sensor device. In the measurement data, there were identified multidimensional patterns, which proved to be specific for the following categories of activities: Night Hours, Outlet Preparation, Food Preparation, Operation Hours, and Cleaning. Their occurrence was recognized by pattern recognition methods with a true positive rate greater than 99%. We demonstrated that the recognition may be based on measurements performed in various locations within the kebab store. Although patterns of the individual categories of activities largely varied between kebab stores, a similar performance of recognition was achieved for all restaurants. The obtained results entitled us to conclude that it is possible to detect activities of QSR, which influenced air quality, with the application of sensor technique and pattern recognition. The proposed approach may be applied to this type of object in general.

## 1. Introduction

The development of Quick Service Restaurants (QSR) is one of the pervasive phenomenon observed in the last several decades [1,2,3]. This advance results from the growing popularity of fast food, which is an important determinant of contemporary mass culture. Currently, fast food restaurants achieved such a level of expansion that they constitute an important branch of the economy. Convenience, good taste, and being economical, in terms of both time and money, are some of the crucial factors acting in favor of the market for fast food and quick-service restaurants. The growth in this field is assisted by the lifestyle expanded around the world and food preferences among generations X, Y, and Z. People do not want to spend a lot of time preparing meals or waiting for meals in restaurants. As a sector of the economy, fast food and quick service restaurants play an important socioeconomic role in meeting food and nutritional requirements, mainly relative to lower and middle-income consumers.

A characteristic element of QSR development is the spread of fast-food outlets [4]. This term denotes premises where food is prepared using standard ingredients and set procedures for cooking. The ready-for-consumption quick meals are sold either on (i.e., at dine-in restaurants) or off premises (as take-away products).

The intensive development of QSR brought increased criticism of these enterprises. The evaluation revealed significant problems that may hamper the growth of this industry. Amongst controversial issues include the quality of meals served in QSR. Generally speaking, it is questionable and presents risks to the health of consumers and constitutes a public health problem. Additionally, scientific research has shown that the exponential growth of the QSR correlates with the obesity epidemic, certainly in the western world. The fast-food industry may contribute to the increasing prevalence of being overweight especially in the low-income population because it ensures the availability of inexpensive meals [5].

The fast-food sector is also criticized for its negative influence on the environment, especially regarding its contributions to greenhouse gas emissions. The institutions involved in environmental protection very often receive complaints against QSR because pollutants emitted from fast food outlets represent a great contributor to outdoor air pollution levels [6]. They cause a nuisance not only to the residents in the immediate vicinity but also to customers and workers within the premises. Indoor air has also become a matter of public concern since it is essential for ensuring a healthy and comfortable work environment and protecting the health of restaurant personnel and visiting customers from exposure to harmful air pollutants [7,8,9]. This interest is enhanced by the fact that people are more aware of the air inside restaurants containing harmful substances. Pollutants emitted by fast food outlets are most often in the form of odors [10]; greasy fume and fallout; fine particulate matter as both solid and aerosol material [11,12]; volatile organic compounds (VOCs) [13]; and toxins. They can significantly impact human health, causing, e.g., skin problems, headaches, respiratory diseases, etc. Benzo(a)pyrene, formaldehyde, and acetaldehyde are classified as known or probable carcinogens.

The impact of QSR on the environment depends on various factors. Many activities and operations that take place at these premises contribute to the pollution generation process. These include storing and handling raw materials; food preparation (e.g., de-boning, grinding, mincing, pureeing, or cutting of foodstuffs); cooking food products for sale to the consumers (e.g., boiling, braising, roasting, frying, barbecuing, etc.); drying food; packaging final products for sale to the public (e.g., takeaway food); selling food products to the public; cleaning; using chemicals; accumulating, storing and disposing of food waste and food-preparation waste; transporting waste from the food outlet. Each of these operations is a source of various, sometimes specific emission. Their impact on people and the environment is versatile. The emissions from fast food outlets include a wide spectrum of chemicals: carbon monoxide (CO); carbon dioxide (CO_2_); nitrogen oxides (NO*_x_*); polycyclic aromatic hydrocarbons (PAHs); volatile organic compounds VOCs (aldehydes are especially important); particulate matter PM (including particles smaller than 100 nm in diameter; particulate matters smaller than 2.5 μm in diameter); liquid oil droplets; water vapor [14,15,16].

From the perspective of air pollution management, it is important to know how sensitive the air quality is to the factors that have an influence on it [17,18,19]. However, it is also essential to recognize when, how often, and for how long these factors are active. Such information can be obtained using different methods, which present the various potentials for application. However, new strategies are still required. In this paper, we want to propose the approach based on sensor measurements of various parameters of indoor air and data analysis using pattern recognition. Our attention was focused on the identification of activities affecting air quality that occur in fast food outlets at different times during the day.

## 2. Experimental Part

### 2.1. Objects

The study was carried out in kebab stores that represent a considerable fraction of fast-food outlets in Poland. Five stores, which operate in a highly populated Polish city, accepted the invitation and joined the experiment. The basic characteristics of these kebab stores are provided in Table 1. The following aspects were taken into account: the type of store, the size of space, its layout, kinds of food offered, food preparation technique, grilling techniques, kind of dishes used, operation hours, peak hours, and throughput.

**Figure 1:**
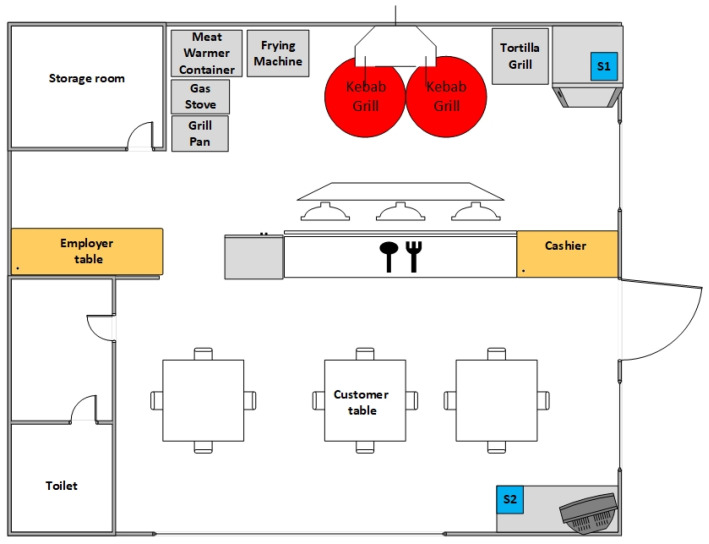
The layout of an exemplary kebab store (kebab 5, see Table 1), including the location of sensor devices (Tables S1 and S2).

As shown in Table 1, the stores were similar in most aspects. They were very limited in terms of size, which is characteristic of takeaway restaurants. The investigated kebab stores had floor areas between 6 and 62 m^2^. Typically, the store occupied one room, as shown in Figure 1. In that room, there were distinguished sub-areas having different functionalities. Usually, they were as follows:

1. The food preparation zone (place for cutting, meat grills, chips frying utensils, and sink) located at the back of the premises;
2. The customer desk located in the middle of the kebab store;
3. The dining area (if it exists) located at the front of the premises.

The stores offered similar kinds of food. The basic ingredients of the dishes were the meat: beef and chicken as well as many kinds of vegetables. Moreover, drinks were served, such as tea, yogurt, and canned drink. Certain differences between kebab stores were associated with the food preparation process. In some cases, the meat was prepared (spicing and marinating) on site. In other cases, ready-made meat was delivered to the store. The majority of stores prepared the meat approximately every 30–45 min or when the meat warmer container was empty. The minority acted upon the order. Kebabs operated from early morning to late evening hours. Throughput was from 40 to 140 customers per day.

### 2.2. Measurements

#### 2.2.1. Equipment

**Figure 2:**
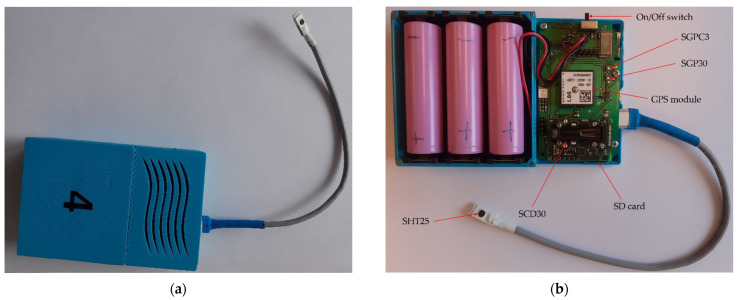
The multi-sensor device applied in the study: (**a**) the external appearance and (**b**) the internal structure: sensor module SHT25 includes high accuracy temperature and relative humidity sensors; sensor module SCD30 includes high accuracy carbon dioxide sensor as well as temperature and relative humidity sensors; sensor module SGPC3 includes indoor air quality sensor for measuring TVOC; sensor module SGP30 includes indoor air quality sensor for measuring TVOC. The size of the device is 12 × 8 × 2.5 cm.

The measurement setup played an important role in our work. The multi-sensor devices were applied to perform the measurements in kebab stores. The following parameters were measured: temperature; relative humidity; pressure; gas sensor response to total volatile organic compounds (TVOC); the equivalent concentration of CO_2_. The device used in this study was developed at Wroclaw University of Science and Technology. It was designed to perform continuous monitoring of indoor air at the site. The instrument was self-contained and battery-powered (see Figure 2). Its size was small (12 × 8 × 2.5 cm). The instrument operates automatically and is unattended and unnoticeable. It consists of a set of sensors. In this study, they were used to determine various characteristics of the environment inside kebab stores.

The multi-sensor device was fitted with the following:

- Sensor module SHT25, which includes high accuracy temperature and relative humidity sensors. It was mounted in the external probe of multi-sensor device (see, Figure 2);
- Sensor module SCD30, which includes high accuracy carbon dioxide sensor. It additionally measures temperature and relative humidity;
- Sensor module SGPC3, which includes indoor air quality sensor for measuring TVOC;
- Sensor module SGP30, which includes indoor air quality sensor for measuring TVOC.

The detailed characteristics of sensors that were used in multi-sensor device are presented in Table 2 [20].

Diffusive gas sampling was used in measurement equipment. The instrument was additionally equipped with a GPS module, which allows it to determine its location. The measurement results were recorded with a temporal resolution of two seconds. In the data file, they were ordered by time. The measurement data were collected on an SD card. A new data file was formed whenever the device was switched on. The instrument requires minimum maintenance, which includes periodic battery exchange and data download. In our study, one set of batteries was sufficient for five days of continuous measurements. The data download frequency was scheduled under the measurement plan.

#### 2.2.2. Location of the Measurement Points and Temporal Aspects of the Measurements

In each kebab store, two measurement points were chosen. One of them was located in the food preparation zone and the other was located in the dining area/customer zone. It can be assumed that activities occurring in these places have different impacts on air quality and people. One sensor device was placed at each measurement point, and the measurements at two points were performed simultaneously. In each kebab store, the indoor air was monitored continuously for five days. Different kebabs were approached in sequence. The entire study took about one month to complete.

#### 2.2.3. Measurement Procedure

The following procedure was applied to perform an indoor air monitoring session in a single kebab store:

1. A lab test of sensor devices (cleaning, connections check, test measurement run, data recording and download check, data completeness check, and battery charge);
2. Measurement session initialization:
  - Delivery of two sensor devices to a kebab store;
  - Measurement points selection;
  - Placement of sensor devices;
  - Switching the devices on; quick check confirming their operation;
  - Noting down the time of measurement session start;
  - Giving kind instructions to the kebab employees (request of not interfering with the measurement devices).
3. Continuous indoor air monitoring for five days (devices remain at the measurement points and they operate unattended);
4. Routine checks and maintenance. They were aimed at visual inspection of the measurement points, batteries exchange in the device, download of the measurement data from the memory card, data verification, and restarting the device to continue monitoring. They were performed once or twice per measurement session;
5. Measurement session completion:
  - Switching off and collection of measurement devices;
  - Giving kind thanks to the kebab owner and staff.

#### 2.2.4. Interview and Observation

The basic characteristics of kebab stores, as well as the information about their functioning, were provided by the kebab store owners and staff. Several interviews were conducted for obtaining this information. Additionally, we performed our observation of kebab functioning on chosen exemplary days.

#### 2.2.5. Categories of Activities

In the course of interviews and observation, the activities that occurred in kebab stores during measurement studies are listed. The activities were grouped into several categories. They were associated with the potential impact of kebab stores on the air quality and people. Five categories of activities were considered. They occurred during the following: night hours; preparation of fast-food outlet to put it into operation; food preparation; operation hours; cleaning of the premises and utilities. The specified categories of activities can be described in the following way:

1. Category—Night Hours (NH). At that time, no activities take place in the kebab store. The premises are closed. No one stays inside. All devices including grills, frying machines, cookers, andventilation devices are switched off. Windows and doors are closed.
2. Category—Preparation of Fast Food Outlet (OP). It includes activities that take place in the period just after opening the store but before starting any operations involving food handling. All sorts of preparatory actions take place. They include checking ingredients, ordering prepared doner kebabs, and preparing the dining area.
3. Category—Food Preparation (FP). It refers to all activities that are aimed at preparing food, which will be served by the fast-food outlet during the entire day. In particular, they are associated with meat and vegetable preparation. Meat preparation involves cutting and marinating chicken breasts and thighs, placing the marinated chicken in a kebab skewer, and wrapping the prepared doner kebab. Vegetable preparation involves washing and cutting vegetables such as tomato, cucumber, olive, and corn, preparing pickled cabbage, lettuce, and carrot. Feta cheese is prepared as well.
4. Category—Operation Hours (OH). This broadest category includes all activities taking place to serve the kebab store clients, on the run, as they arrive. Operation hours are featured by the presence of a various number of clients in the store (except for store 1 which is strictly take away one), including occasions when no one is served. This implies changing the intensity of the kebab operation. Typically, client service takes 10–15 min and it includes frying potatoes, grilling doner kebab, and plating salad, sauce, potato, and sliced kebab. Different orders are reflected in various impacts on air quality and people.
5. Category—Cleaning of Premises and Utilities (CL). This category includes cleaning activities, which are aimed at keeping the kebab place in order and tidy. During cleaning time, all cooking, grilling, and frying utensils are stopped for cleaning and washing. The frying oil is disposed of. Tables, customer desks, and other surfaces are wiped wet. The floor is brushed and wiped. Bins are emptied and cleaned. Dishes are washed. The food which has not been consumed is put in the fridge or the employees will take away it.

The timing of the listed categories of activities differed among kebabs. Additionally, for the individual store, their occurrence and duration were not fixed, as well. They were dependent on the actual opening and closing time as well as the specific circumstances during a particular day.

## 3. Methods of Data Analysis

### 3.1. Pattern Recognition

We examined the possibility of recognizing the categories of kebab activities based on indoor air quality sensing. Activities belonging to different categories had various impacts on air quality. Hence, the information about them is important from the perspective of environmental management.

The pattern recognition approach was applied to achieve the goal of the study. A five-class classification problem was formulated, which consisted in distinguishing five categories of kebab activities, listed in Section 2.2.5. The recognition was based on the measurement data collected using a multi-sensor device. The data were included in the specially constructed feature vectors.

### 3.2. Data

Three sources of input data for pattern recognition models were considered. They were indoor air measurements collected in the following areas: (1) food preparation zone; (2) customer zone; (3) food preparation and customer zones, jointly. The data included in these datasets were assumed to be indicative of different categories of kebab activities. However, they presented information about the patterns of activities from different perspectives. It was expected that the trained classifiers can generalize over the variation of patterns in a particular zone.

### 3.3. Feature Vectors

Four parameters of indoor air were selected for kebab activities recognition. They were as follows: temperature measured using a sensor located in the external probe of the multi-sensor device; relative humidity measured using a sensor located in the external probe of the multi-sensor device; carbon dioxide concentration and the response of sensor SGP30 dedicated to measuring TVOCs. Using these parameters, three different feature vectors were constructed:

1. Feature Vector 1 (FV1) consisted of raw results of a single measurement. It included the values of four selected parameters of indoor air. When proposing this feature vector, the assumption was made that the type of activity occurring inside the kebab store may be recognized based on a single measurement of the multi-sensor device. With FV1 as the input, the classification system obtained information about the momentary magnitude of the indoor air parameters. The major advantage of this feature vector was the simplicity of data pre-processing and the possibility of performing nearly real-time predictions.
2. Feature Vector 2 (FV2) consisted of raw results of one-minute-long monitoring. The feature vector was composed of raw values of four selected indoor air parameters collected in this period. When proposing such a feature vector, the assumption was made that different categories of kebab activities may be recognized based on a short time series of measurements performed using a multi-sensor device. With FV2 as the input, the classification system obtained information about the magnitude of the measured parameters and their temporal change. This feature vector contained exhaustive information.
3. Feature Vector 3 (FV3) included the spread of the results obtained during one-minute-long monitoring. The feature vector was composed of standard deviations of the selected indoor air parameters in this period. When proposing such a feature vector, the assumption was made that different categories of kebab activities may be recognized based on a short time series of measurements performed using the multi-sensor device, such as in case 2. However, the important information is contained in the alteration of indoor air parameters. With FV3 as the input, the classification system was presented only with the indication of the change of indoor air parameters, excluding their magnitude. This feature vector contained compressed information.

### 3.4. Classifier

The classification process was performed using Random Forest Classifier (RFC). RFC is a supervised machine learning technique for classification. The random forest consists of many decision trees. It combines the simplicity of decision trees with the flexibility to vastly improve accuracy [21].

There are several steps to operating a random forest classifier algorithm. Firstly, the data are randomly selected from the original data set with replacement. This process is referred to as bootstrapping. Secondly, a decision tree is created using the bootstrapped dataset, which only considers a random subset of the independent variables at each step. Then, the previous steps are repeated to result in a wide variety of trees. The variety causes random forests to be more effective than the individual decision tree. After that, each decision tree is trained independently to obtain the result. The final output is determined by the voting system and includes all results. The majority vote then becomes the final decision. This voting system is known as aggregation. Bootstrapping combined with aggregation refers to bagging [22,23]. Random Forest can decrease overfitting through bagging technique and random feature selection [24]. It results in high accuracy, even in the case of working with large data sets. The structure can be easily saved for future use [25].

### 3.5. Performance Assessment

Upon classification model building, the entire measurement data set was divided into the training set (70%) and the testing set (30%). Stratified random sampling was applied for assigning the data to these sets. The strata were the measurement data associated with the occurrence of a particular category of activities in the kebab store. The classifier was trained using training data and it was tested using the testing data.

Classification performance was evaluated using a 5 × 5 confusion matrix, which was developed for the test set. The confusion matrix served to examine the misclassification structure in detail. Additionally, the true positive rate (TPR) was determined for each category of kebab activities. The true positive rate is the probability that an actual positive will test positive. It indicates the percentage of successful classifications within a particular class. TPR was applied to express pattern recognition results in the synthetic form, which allows for the comparison between multiple variants of classification.

## 4. Results

### 4.1. Patterns of Kebab Activities Categories

**Figure 3:**
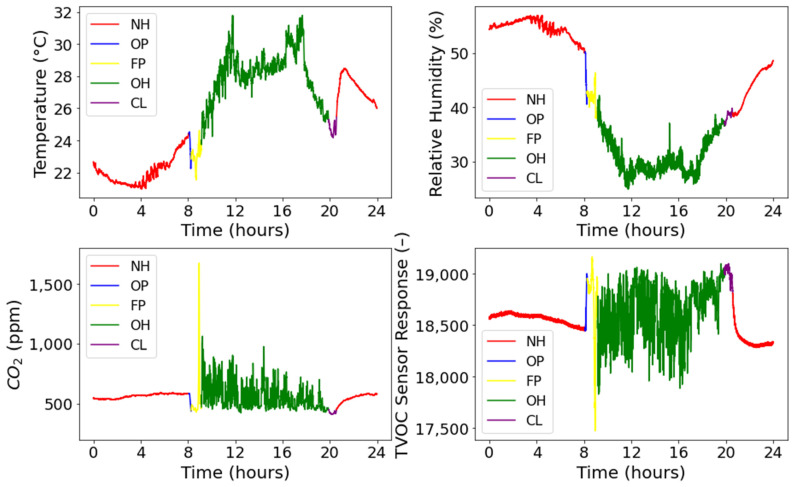
Time series of indoor air parameters: temperature, relative humidity, CO_2_ concentration, and the indication of TVOC content. They were recorded in the food preparation zone of the exemplary kebab store during 24 h monitoring. Colors indicate the fragments of data series associated with the following categories of kebab activities: NH—Night Hours; OP—Outlet Preparation; FP—Food Preparation; OH—Operation Hours; CL—Cleaning.

The exemplary results of kebab air monitoring are shown in Figure 3. They represent the typical features of the entire set of measurement data that were collected during the study. The displayed parameters are air temperature; relative humidity; CO_2_ concentration; and the indication of TVOC concentration. The period of 24 h was chosen to visualize the distribution of categories of kebab store activities in time, during an average working day. The time series in Figure 3 were colored for showing their fragments associated with the occurrence of different categories of kebab activities. The average duration of these fragments was additionally summarized in Table 3. Based on Figure 3, the individual categories of kebab activities had different times of occurrence. As shown in Table 3, Night Hours was the longest-lasting category (11 h and 36 min). The second long-lasting category was Operation Hours (10 h and 45 min). Three other categories were observed in much shorter periods. They were the following: Cleaning—41 min; Outlet Preparation—6 min; Food Preparation—52 min. Due to the shorter period of occurrence, these categories were underrepresented in the data sets used for pattern recognition model development. The unequal representation of classes in the training data sets results in an unbalanced classification problem. In such cases, the less numerous categories are usually poorly recognized compared with the well-represented categories, unless their patterns are highly distinctive.

In Figure 3, the correspondence between the type of activities that take place in kebabs and the behavior of indoor air parameters at that time was observed. As shown, the magnitudes as well the variation of parameters were related to the occurrence of various categories of kebab activities.

At night, indoor conditions in the stores were stable and mostly undisturbed. Hence, the essential feature of the category Night Hours (red line in Figure 3) was a very small variation of indoor air parameters. This particularly referred to CO_2_ and TVOC. The concentrations of these compounds were relatively small due to the absence of people and lack of open-flame cooking in the store at night. They are both sources of CO_2_ and TVOC emissions. Additionally, the category Night Hours was associated with high air humidity in the store. The temperature usually stayed at a low level.

Another distinctive category of kebab activities was the Operation Hours (green line in Figure 3). It was featured by the high temperature in the store. Human presence as well as frying and cooking operations contributed to the increase in indoor temperature. The rising temperature was also related to the external, meteorological factors because the experiment was conducted during the summertime. Operation Hours were indicated by a relatively high concentration of CO_2_ and TVOC in kebab air. Additionally, these parameters displayed a significant temporal variation. During operation hours, the CO_2_ and TVOC sources in the store remained active and the intensity of emission was altered. Food was prepared on demand; therefore, cooking and frying devices operated irregularly. Additionally, the customer load was variable.

The measurement data pattern of the category called Food Preparation (yellow line in Figure 3) also has specific features. These were the peeking concentrations of CO_2_ and VOCs. This behavior was caused by the intensive grilling process, which was usually conducted in the morning, for 15 to 30 min, to fill the meat warmer container. Meat grilling also contributed to the temperature increase, which was observed shortly after the Outlet Preparation phase. Additionally, preparing vegetables generated VOCs emission and it was also a source of water vapor emission, causing moisture content to increase in the air.

The category Cleaning (violet line in Figure 3) displayed yet another characteristic in measurement data. The highest level of TVOC in kebab air was noted, which could have resulted from the application of cleaning products. The humidity level increased, mostly because of washing. The employee wiped the tables, all kinds of surfaces, and the floor. On the other hand, due to the inactive combustion source (grilling process), the temperature and concentration of CO_2_ decreased compared with conditions specific to the other categories of kebab activities.

The least distinctive category was Outlet Preparation (blue line in Figure 3). In Figure 3, the occurrence of this category may be observed as the break in the tendency associated with night hours. The specific feature of Outlet Preparation was the drastic increase or decrease in all measured parameters. This change was caused by opening the store and letting ambient air inside. The duration of the Outlet Preparation phase was short. Its pattern in the measurement data was most similar to the one of Night Hours.

**Figure 4:**
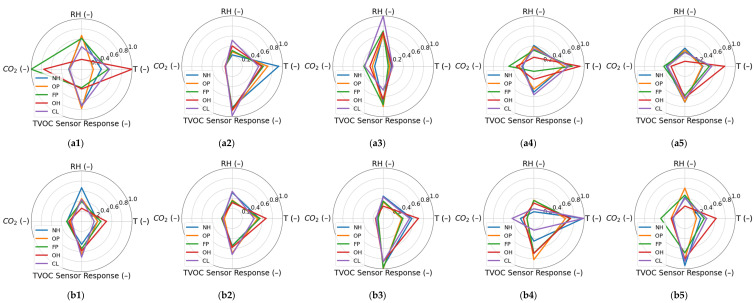
Patterns of kebab activities categories in the domain of indoor air parameters recorded in two measurement point locations: (**a1**–**a5**) food preparation area in kebab 1 to 5; (**b1**–**b5**) customer zone in kebab1 to 5. The parameters were scaled. TVOC sensor response was divided by 500 and CO_2_ concentration was divided by 50 to obtain a similar range with relative humidity and temperature. Then, the average values of the measured parameters from five kebabs including two locations in each of them were jointly scaled from 0.1 to 1. Categories of kebab activities are indicated as follows: NH—Night Hours; OP—Outlet Preparation; FP—Food Preparation; OH—Operation Hours; CL—Cleaning.

In this study, we assumed that different categories of kebab store activities resulted in different patterns of indoor air parameters. Figure 4 compares the patterns of kebab activities categories reflected in the measurement data collected during the measurement experiment. For each kebab, there were considered patterns associated with the food preparation zone and the customer zone. The patterns were obtained by averaging the values of parameters measured upon the occurrence of a particular activity. The data were scaled to account for differences in the range of values of the individual parameters. As shown in Figure 4, the occurrence of different categories of kebab store activities resulted in different measurement data patterns. However, based on our results, kebab activity patterns were largely kebab-specific. This fact was revealed by the comparison of patterns derived from the data collected in distinct kebab stores at similarly located measurement points, as shown in Figure 4. Additionally, the patterns of activities were not spatially homogeneous within a particular kebab store. It is shown in Figure 4 by the comparison of patterns derived from the measurement data collected in the food preparation zone and the customer zone. The patterns presented in Figure 4 justify the development of pattern recognition models focused on the classification and recognition of kebab activity categories. However, given these results, it is assumed that pattern recognition models shall be developed individually for each kebab and they should be based on the data collected in the individual measurement location. The option of combining results collected in multiple measurement points was considered additionally.

### 4.2. Recognition of Kebab Activities Categories

The performance of kebab activities classification was summarized in Table 4, Table 5 and Table 6. They refer to three kinds of feature vectors used for pattern recognition and consist of raw results of a single measurement (FV1, Table 4), raw results of one-minute long monitoring (FV2, Table 5), and the spread of the results obtained during one-minute long monitoring (FV3, Table 6). The obtained results of classification indicate that kebab activities categories may be effectively recognized based on measurements of indoor air with a multi-sensor device. The indoor air parameters such as temperature, relative humidity, CO_2_ concentration, and the indication of TVOC content hold relevant information. The best results of recognition were attained using FV2 (TPR ≥ 99.93%) (see Table 5). The slightly weaker performance was achieved with FV1 (TPR ≥ 99.37%) (see Table 4). The results were least satisfactory in the case of FV3 (TPR ≥ 88.61%) (see Table 6). The classification performance evaluation shows that the magnitude (FV1), as well as the variation (FV3) of indoor air parameters are good descriptors of kabab activities. Still, the most complete information was obtained when utilizing both of them as the basis of pattern recognition (FV2).

**Figure 5:**
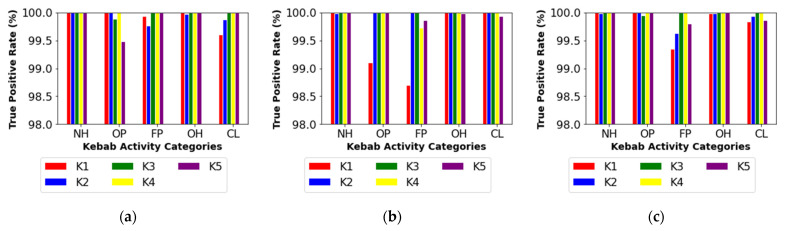
True positive rate (TPR) for the recognition of the individual categories of kebab activities using a one-minute-long measurement (feature vector 2) performed in: (**a**) food preparation zone, (**b**) customer zone, and (**c**) food preparation zone or customer zone. Categories of kebab activities are indicated as follows: NH—Night Hours; OP—Outlet Preparation; FP—Food Preparation; OH—Operation Hours; CL—Cleaning.

In Figure 5, we present the detailed results of kebab activity recognition using the best approach, which consisted in applying FV2 as the basis of classification. Color bars display the true positive rate for recognizing the individual categories of activities. It could be observed that pattern recognition performance was related to the source of measurement data. As shown in Figure 5a, measurements performed in the food preparation zone were the imperfect predictors of the Outlet Preparation category. The respective confusion matrices (see Supplementary Material) show that Outlet Preparation was mistaken for Night Hours (kebab 2 and kebab 4) or it was mistaken for Food Preparation (kebab 1). Based on Figure 5b, the use of measurements performed in the customer zone resulted in weaker recognition of patterns representing categories of Food Preparation and Cleaning. The respective confusion matrices (see Supplementary Material) show that Food Preparation and Cleaning were mistaken for Operation Hours. All over, we see that the activities that take place predominantly in a particular space create the most readable pattern in the air of that space. While Outlet Preparation was more visible in the customer zone, Food Preparation and Cleaning were better recognized from the data collected in the food preparation zone. Combining the measurement data from both locations resulted in the improved recognition of the categories Outlet Preparation and Cleaning. Regarding Food Preparation, the shift in performance was kebab-dependent. Therefore, from our results, it was not obvious that the approach consisting in combining measurement data collected in several locations had been advantageous given the improving recognition of kebab activity categories.

## 5. Conclusions

The objective of this study was to examine the possibility of detecting activities occurring inside Quick Service Restaurants that influence air quality, with the application of sensor technique and pattern recognition.

The presented analysis was based on an experimental study, which involved indoor air monitoring in kebab stores in a highly populated Polish city. Based on measurement results, five categories of kebab activities were distinguished, which had different impacts on air quality. They were the following: Night Hours, Outlet Preparation, Food Preparation, Operation Hours, and Cleaning. The pattern recognition approach was employed to recognize these categories based on the following quantities measured indoors: temperature, relative humidity, CO_2_ concentration, and TVOC content.

The results of classification model testing showed that the efficiency of recognition of kebab store activities categories exceeded 99% (true positive rate) for the individual restaurant.

We demonstrated that recognition may be based on measurements performed in various locations within the kebab store. However, our results show that the activities that mostly occur in a particular zone of the restaurant produce more distinctive patterns in the air of that zone.

Based on our analysis, the patterns of distinct categories of activities largely varied between kebab stores. However, a similar performance of recognition was obtained for all restaurants. This result entitles us to conclude that it is possible to detect activities occurring inside QSR that influence air quality with the application of sensor technique and pattern recognition. The proposed approach may be applied to this kind of object in general.

In future studies, we plan to develop a method to estimate quantitatively the impact of distinct QSR activities on air quality. The proposed approach will be based on multi-sensor measurements. In our opinion, this work as well as its planned continuation may be of interest to environmental engineers, QSR managers, and municipalities.

## Figures and Tables

**Table 1:** Basic characteristics of kebab stores, which were involved in the study.

Parameter	Kebab 1	Kebab 2	Kebab 3	Kebab 4	kebab 5
Type of the store	Take away store	QSR	QSR	QSR	QSR
Volume	2 m × 3 m × 2 m	7 m × 6 m × 2.5 m	8 m × 4 m × 2.5 m	6 m × 6 m × 2.5 m	7 m × 5 m × 2.3 m
Meat (Chicken)	The chicken meats are supplied from the meat supplier. The chicken has been marinated with Mediterranean spices and then wrapped before being delivered to the kebab store	The chicken is prepared at home (homemade).	The chicken is prepared by the owner in the kitchen.	Same as Kebab 1	Same as Kebab 1
Meat (Beef)	Same as chicken meat	There is no beef kebab in this restaurant	Same as kebab 1	Same as kebab 1	Same as kebab 1
Menu list	Beef kebab Chicken kebab Tortilla Salad French fries	Chicken kebab Tortilla Grilled beef Falafel Yellow rice Salad French fries Baklava	Beef kebab Chicken kebab Tortilla Falafel Salad French fries Baklava	Beef kebab Chicken kebab Tortilla Falafel Salad French fries	Beef kebab Chicken kebab Tortilla Falafel Salad French fries
Number of grillers	2 gas electric grills	2 gas electric grills	3 gas electric grills (Normally only 2 grills are used. However, during Friday and Saturday night, they used 3 grillers.)	2 gas electric grills	2 gas electric grills
Food Preparation Technique	The doner kebabs (chicken and beef) are grilled for 15–30 min to fill the meat warmer container. The steps of preparing orders include: The employee fries the French fries and then seasons them. At the same time, he will arrange the salad and add the sauce to the pita bread. Then, he will put in the heated meat and roll the kebab before grilling it on the tortilla grill.	Generally, food preparation techniques are similar to kebab 1. The differences include: French fries can be substituted with yellow rice The chicken meat will be grilled after customers order their meals. Therefore, it takes about 15–18 min to prepare the food. It takes around 20 min to prepare the grilled beef.	Generally, food preparation techniques are similar to kebab 1. However, the employee will serve the meals on a plate for customers who want to enjoy their food in the restaurant.	Same as kebab 3	Same as kebab 3
Grilling Technique	Doner kebab is cooked by turning it in the face of fire. First 1 h it is low cooked in the fire. The cooked kebab is cut thinly using long doner knife.	Same as kebab 1	Same as kebab 1	Same as kebab 1	Generally, the grilling technique is similar to kebab 1. Meanwhile, the employee sometimes cooks the kebab over high heat producing smoke in grilling area
Operational hours	9.00–22.00	9.00–22.00	Weekdays: 9.00–22.00 Weekend: 9.00–6.00 the next day	Weekdays: 9.00–22.00 Weekend: 9.00–23.00	Weekdays: 10.00–22.00 Weekend: 10.00–0.00
Peak hours	13.00–16.00	13.00–16.00	12.00–13.00 and 1.00–2.00	13.00–16.00	13.00–16.00
Plate used	No plate, only take away	Ceramic plate and small sauce containers	Ceramic plate	Disposable plate	Disposable plate
Staff	1 employee	2 employees	2 employees	2 employees	1 employee
Throughput	60–70 costumers per day	100–140 costumers per day	60–110 costumers per day	60–70 costumers per day	40–50 costumers per day

**Table 2:** The detailed characteristics of sensors that were used in multi-sensor device [20].

Sensor	Measured Parameter	Detection Principle	Measurement Range	Accuracy	Resolution	Repeatability	Long Term Drift
SHT25	Temperature	band gap temperature sensor	−40 to 125 °C	Typ. ±0.2 °C	0.04 °C	±0.1 °C	<0.02 °C/yr
	Relative humidity	capacitive type humidity sensor	0 to 95%RH	±1.8%RH	0.04%RH	±0.1%RH	<0.25 %RH/yr
SCD30	CO_2_	Non Dispersive Infrared (NDIR) measurement technology	0–5000 ppm (PWM)	±(30 ppm + 3% meas. value)	-	±10 ppm	±50 ppm
	Temperature	band gap temperature sensor	−40 °C–70 °C	±(0.4 °C + 0.023 × (T [°C] − 25 °C))	-	±0.1 °C	<0.03 °C/yr
	Relative humidity	capacitive type humidity sensor	0 %RH–95 %RH	±3%RH	-	±0.1%RH	<0.25 %RH/yr
SGPC3	TVOC	metal-oxide gas sensor (chemoresistive measurement principle)	0.3 ppm to 30 ppm ethanol 0 ppm to 1000 ppm ethanol	Typ. 15% of meas. value	Typ. 0.2 % of meas. value	-	Typ. 1.3% of meas. value
SGP30	TVOC	metal-oxide gas sensor (chemoresistive measurement principle)	0.3 ppm to 30 ppm ethanol 0 ppm to 1000 ppm ethanol	Typ. 15% of meas. value	Typ. 0.2 % of meas. value	-	Typ. 1.3% of meas. value

**Table 3:** The occurrence of the distinguished kabab activities categories during an average working day.

Object	Night Hours	Outlet Preparation	Food Preparation	Operation Hours	Cleaning
Kebab 1	11 h and 36 min	5 min	1 h and 13 min	11 h and 13 min	35 min
Kebab 2	11 h and 45 min	41 min	40 min	10 h and 7 min	46 min
Kebab 3	7 h and 13 min	34 min	1 h and 6 min	12 h and 25 min	25 min
Kebab 4	10 h	5 min	33 min	12 h and 56 min	33 min
Kebab 5	8 h and 43 min	7 min	42 min	13 h and 57 min	46 min

**Table 4:** Average TPR associated with the recognition of kebab activities categories based on FV 1.

Object	Location of the Measurement Point		
	Food Preparation Zone	Customer Zone	Food Preparation Zone or Customer Zone
Kebab 1	99.37%	99.89%	99.59%
Kebab 2	99.83%	99.45%	99.60%
Kebab 3	99.95%	99.91%	99.92%
Kebab 4	99.98%	99.96%	99.95%
Kebab 5	99.95%	99.75%	99.80%

**Table 5:** Average TPR associated with the recognition of kebab activities categories based on FV 2.

Object	Location of the Measurement Point		
	Food Preparation Zone	Customer Zone	Food Preparation Zone or Customer Zone
Kebab 1	99.93%	99.98%	99.96%
Kebab 2	100.00%	99.98%	99.98%
Kebab 3	100.00%	100.00%	99.99%
Kebab 4	100.00%	99.99%	99.99%
Kebab 5	100.00%	99.99%	99.99%

**Table 6:** Average TPR associated with the recognition of kebab activities categories based on FV 3.

Object	Location of the Measurement Point		
	Food Preparation Zone	Customer Zone	Food Preparation Zone or Customer Zone
Kebab 1	89.82%	94.57%	91.37%
Kebab 2	94.25%	92.68%	90.00%
Kebab 3	88.61%	93.08%	89.02%
Kebab 4	90.91%	93.54%	91.45%
Kebab 5	91.18%	93.29%	90.85%

## Data Availability

The data are not publicly available and are available upon request from the owners of the premises where the measurements were performed.

## Supplementary Materials

The following supporting information can be downloaded at: https://www.mdpi.com/article/10.3390/s22114056/s1, Table S1: Confusion matrices for classification of kebab activity categories, using Feature vector 2 as the input of the classifier. Table S2: Confusion matrix for classification of kebab activity categories, based on measurement data collected in Kabab 1, in customer zone. Feature vector 2 was used as the input of the classifier. Table S3: Confusion matrix for classification of kebab activity categories, based on measurement data collected in Kabab 1, in food preparation zone and in customer zone, jointly. Feature vector 2 was used as the input of the classifier.
